# Effect of timeframes to define long term conditions and sociodemographic factors on prevalence of multimorbidity using disease code frequency in primary care electronic health records: retrospective study

**DOI:** 10.1136/bmjmed-2022-000474

**Published:** 2024-02-13

**Authors:** Thomas Beaney, Jonathan Clarke, Thomas Woodcock, Azeem Majeed, Mauricio Barahona, Paul Aylin

**Affiliations:** 1 Department of Primary Care and Public Health, Imperial College London, London, UK; 2 Department of Mathematics, Imperial College London, London, UK

**Keywords:** Epidemiology, Healthcare Disparities, Public health, Primary health care

## Abstract

**Objective:**

To determine the extent to which the choice of timeframe used to define a long term condition affects the prevalence of multimorbidity and whether this varies with sociodemographic factors.

**Design:**

Retrospective study of disease code frequency in primary care electronic health records.

**Data sources:**

Routinely collected, general practice, electronic health record data from the Clinical Practice Research Datalink Aurum were used.

**Main outcome measures:**

Adults (≥18 years) in England who were registered in the database on 1 January 2020 were included. Multimorbidity was defined as the presence of two or more conditions from a set of 212 long term conditions. Multimorbidity prevalence was compared using five definitions. Any disease code recorded in the electronic health records for 212 conditions was used as the reference definition. Additionally, alternative definitions for 41 conditions requiring multiple codes (where a single disease code could indicate an acute condition) or a single code for the remaining 171 conditions were as follows: two codes at least three months apart; two codes at least 12 months apart; three codes within any 12 month period; and any code in the past 12 months. Mixed effects regression was used to calculate the expected change in multimorbidity status and number of long term conditions according to each definition and associations with patient age, gender, ethnic group, and socioeconomic deprivation.

**Results:**

9 718 573 people were included in the study, of whom 7 183 662 (73.9%) met the definition of multimorbidity where a single code was sufficient to define a long term condition. Variation was substantial in the prevalence according to timeframe used, ranging from 41.4% (n=4 023 023) for three codes in any 12 month period, to 55.2% (n=5 366 285) for two codes at least three months apart. Younger people (eg, 50-75% probability for 18-29 years *v* 1-10% for ≥80 years), people of some minority ethnic groups (eg, people in the Other ethnic group had higher probability than the South Asian ethnic group), and people living in areas of lower socioeconomic deprivation were more likely to be re-classified as not multimorbid when using definitions requiring multiple codes.

**Conclusions:**

Choice of timeframe to define long term conditions has a substantial effect on the prevalence of multimorbidity in this nationally representative sample. Different timeframes affect prevalence for some people more than others, highlighting the need to consider the impact of bias in the choice of method when defining multimorbidity.

**WHAT IS ALREADY KNOWN ON THIS TOPIC**

- Multimorbidity is defined as the presence of two or more long term conditions
- Large differences in which conditions are included in this definition exist
- How the time periods used to define a long term condition based on the frequency of disease codes in electronic health record data affect multimorbidity prevalence is unknown

**WHAT THIS STUDY ADDS**

- Comparing five timeframes to define 41 of a set of 212 long term conditions, where presence of a single disease code in the electronic health record might represent an acute condition, substantial variation was shown in the prevalence of multimorbidity
- Multimorbidity prevalence ranged from 41.4% (three codes required within any 12 month period) to 73.9% (if a single code was sufficient for diagnosis); using diseases recorded by clinicians in the electronic health record as active problems, multimorbidity prevalence was only 35.2%
- Younger people, people in some minority ethnic groups, and people living in areas of lower socioeconomic deprivation were more likely to be re-classified as not multimorbid under timeframes requiring more than one code

**HOW THIS STUDY MIGHT AFFECT RESEARCH, PRACTICE, OR POLICY**

- Substantial variation was noted in multimorbidity prevalence according to the timeframe used to define a long term condition, highlighting the challenges in the direct comparison of estimates between studies
- Recommendations are provided for researchers applying different timeframes for counting long term conditions using electronic health record data
- Definitions of long term conditions requiring multiple codes may introduce bias, and researchers should provide a rationale for their choice of timeframe and consider sensitivity analyses to explore the impact on different patient groups

## Introduction

Multimorbidity is defined as the co-existence of two or more long term conditions in the same person and is a priority for health systems globally.^1 2^ The number of people living with multimorbidity is growing, partly due to population ageing, with a recent UK based study finding an increase in prevalence in adults from 31% in 2004 to 53% in 2019 and with higher incidence associated with increased socioeconomic deprivation.^3^ Although most studies define multimorbidity as the presence of two or more long term conditions,^4^ no consensus exists as to which conditions should be included. One systematic review identified a range from two to 285 separate conditions included in multimorbidity measures,^4–6^ making comparison between prevalence estimates challenging.

A further challenge to estimating multimorbidity prevalence is to distinguish acute from chronic presentations, particularly when using routinely collected electronic health record data. Most commonly, conditions are characterised as ‘chronic’ based on duration,^6^ ranging from at least three or 12 months, to lifelong.^6–8^ Although for many conditions, such as ischaemic heart disease, a single diagnostic code indicates a lifelong condition, for others, such as gastritis, a single code may not differentiate an acute episode from chronic disease, or active from inactive disease. Researchers using electronic health record data have used different approaches to distinguish these: some studies have required multiple entries of a diagnostic code spaced over time for particular diseases, while others have made use of prescriptions of related medications within a specific time period.^3 9^ A potential alternative to using occurrences of diagnostic codes is to use conditions that a clinician has classified as being a problem within the healthcare record. These conditions are recorded separately from clinical diagnoses and are used by clinicians as a summary of a patient’s active clinical problems.^10^

Whether the prevalence of multimorbidity is substantially affected by the choices over the use of diagnosis or problem codes is unclear, as is the timeframe for determining whether a person has a long term condition and whether definitions may impact some groups of people differently. The aim of this paper, therefore, is to determine the extent to which different strategies that are used to define multimorbidity in the primary care electronic health record affect the prevalence of multimorbidity overall and between different demographic groups. We used a range of timeframes from previous multimorbidity research to define long term conditions, and applied these to a large, nationally representative sample of patients in the primary care electronic health record in England.

**Figure 1:**
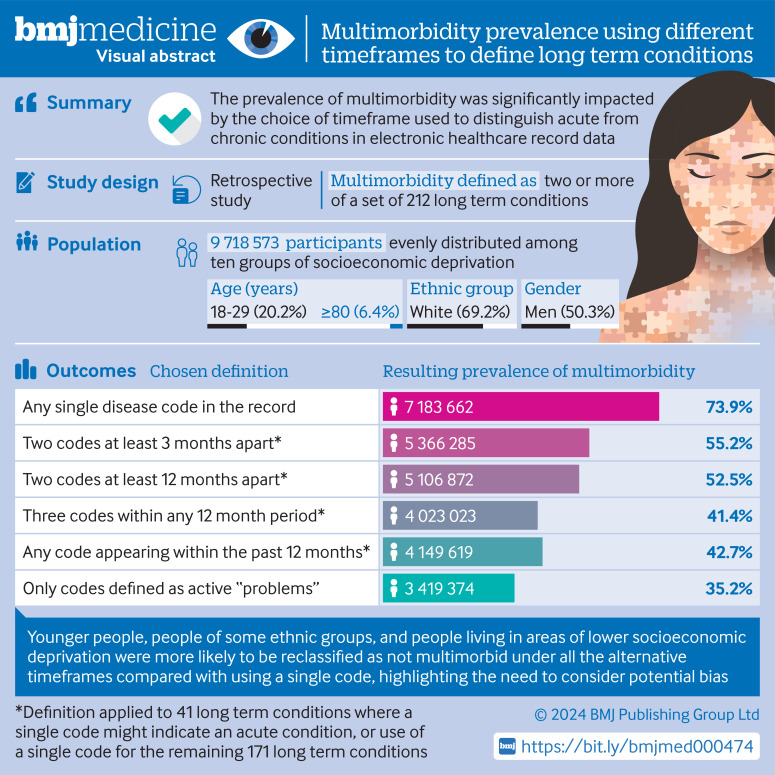
Visual abstract

Figure 1 shows the visual abstract.

## Materials and methods

### Data source

We used data provided from the Clinical Practice Research Datalink Aurum dataset, which includes routinely collected, patient level data from general practices using EMIS health web software in England.^11^ We included all patients who were deemed research acceptable (a marker used by the Clinical Practice Research Datalink for patients meeting specific quality criteria^12^) registered to a general practice on 1 January 2020 and aged 18 years or older with at least one year of continuous practice registration. Observation data for each patient are recorded in the form of Medcodes, along with the date of observation and entry date. Any observation codes occurring after the 1 January 2020 were excluded and we included all historical observation codes. Data were linked to the 2019 Index of Multiple Deprivation of a patient’s area of residence (categorised into tenths), which was used as a patient level marker of socioeconomic deprivation.^13^

### Diagnostic codes and data cleaning

Multimorbidity was defined as the presence of two or more chronic conditions from a set of 212 conditions.^4^ We included 211 conditions selected by Head and colleagues, based on a set of 308 disease phenotypes developed by Kuan and colleagues.^3 14^ We reviewed these code lists and made changes for diabetes disease lists (online supplemental appendix) and also included "chronic primary pain" as a new condition. In the study by Head and colleagues, most conditions were defined by a diagnostic code ever being recorded, but for some conditions, the authors required three occurrences of a code within a 12 month period. Two clinicians (TB and JC) reviewed the codes and reassigned 41 diseases to require multiple codes where a single code might otherwise indicate an acute condition (see online supplemental appendix for further details and rationale). For diabetes, we assigned the most likely diabetes type (type 1, type 2, or other/unspecified) based on the frequency of codes. Further information on the long term condition categories and cleaning of ethnic group codes are given in the online supplemental appendix. Our code lists are available from: https://tbeaney.github.io/MMclustering/

10.1136/bmjmed-2022-000474.supp1 Supplementary data

### Disease definitions

**Figure 2:**
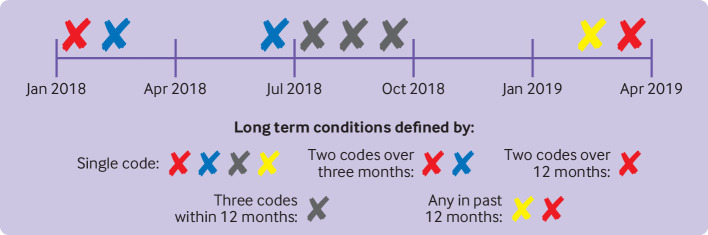
Schematic diagram of assignment of a long term condition for a hypothetical patient under each alternative definition. Each cross represents a code for a condition in a single patient s electronic health record timeline from January 2018 to April 2019, with the colour representing the disease. The single code means a single code was sufficient for diagnosis results in all four long term conditions being counted. Two codes over three months and two codes over 12 months required at least two codes separated by at least three months and 12 months, respectively. Three within 12 months’ requires three codes occurring within a 12 month time window. Any in past 12 months requires at least one code occurring in the past 12 months (measured in the 12 months from 1 January 2019 to 1 January 2020. Diseases defined by problems not included here

The number of chronic conditions for each person was calculated as of the 1 January 2020 under each of the following five definitions (figure 2). These definitions were selected to reflect the variety used in previous multimorbidity research and definitions of chronic conditions:

1. Any disease code in the record for all 212 conditions, which was used as the reference definition (single code);^14^
2. two codes at least three months apart for the 41 conditions requiring multiple codes,^7^ or a single code for the remaining 171 conditions;
3. two codes at least 12 months apart for the 41 conditions requiring multiple codes,^7 8^ or a single code for the remaining 171 conditions;
4. three codes within any 12 month period for the 41 conditions requiring multiple codes,^3^ or a single code for the remaining 171 conditions; and
5. any code appearing within the past 12 months for the 41 conditions requiring multiple codes,^9^ or a single code for the remaining 171 conditions.

We also compared a definition of multimorbidity only including conditions defined as active problems (referred to here as problems). Diagnostic codes entered into the record can be marked by clinicians as problems, which then appear on a patient’s summary during a consultation.^11^ These may be more visible to the clinician than historical diagnostic codes, which would require manual searching through the electronic notes and are also pulled across onto referral letters.^10^ Problems are recorded as either active or past problems and can be marked as ended if no longer active, with the end date recorded.^11^ We included only those problems that were active, that is, those that did not have an associated end date before 1 January 2020 and were marked as active. The problem definition was applied to all 212 long term conditions and not only the 41 requiring multiple codes. Further details are given in the online supplemental appendix page 10.

### Statistical analysis

We calculated the prevalence of patients with one, two, or three or more chronic conditions based on each definition, stratified by age group, gender, ethnic group, and socioeconomic deprivation. Age (years) was categorised into 18-29, 30-39, 40-49, 50-59, 60-69, 70-79, and 80 or more. For gender, we used the categories defined in Clinical Practice Research Datalink, as male, female, or indeterminate (where gender cannot be classified as male or female). No data for age or gender were missing due to our inclusion criteria and Clinical Practice Research Datalink's definition of research acceptable patients. In all analyses, missing data were assigned as categories to ensure no patients were excluded.

To calculate associations with the expected probability of a change in having multimorbidity under each definition, we used mixed effects logistic regression. We included only patients with multimorbidity defined using the single code definition and created a binary outcome variable as 1 if multimorbidity status changed and 0 if multimorbidity status did not change, compared with the single code definition. We included age, gender, ethnic group, and Index of Multiple Deprivation as covariates in the models and random intercepts for general practice to account for clustering and potential coding differences between practices. We then calculated the marginal probability of a change in multimorbidity status overall, and separately for each covariate, holding other covariates at their mean values and assuming random effects of zero.

To calculate associations with the change in expected total count of conditions under each definition compared with the single code definition, we used mixed effects negative binomial regression. A negative binomial model was chosen because the conditional variance was found to be significantly larger than the conditional mean for each model (P<0.001) making a Poisson model unsuitable.^15^ Models included the same covariates and random effects as in the mixed effects logistic regression models. We then calculated the marginal expected change in the count of long term conditions overall and separately for each covariate, assuming other covariates in the model were held at their mean values and random effects of zero. All regression models assumed the single code definition as the reference group, given that this method will always result in the highest estimated prevalence. Further details, including equations for both models, are given in the online supplemental appendix.

Python version 3.10.6 and Pandas version 1.4.3 were used in data processing and plots and Stata version 17.0 was used for regression analyses.

## Results

We included 9 718 573 adults aged 18 years or older and registered in the Clinical Practice Research Datalink Aurum on 1 January 2020 in the study. Table 1 shows the demographic characteristics of patients included. 20.2% of participants were aged 18-29 years and 6.4% were 80 years or older. Gender was almost equally split between women and men (49.7% and 50.3%, respectively) with less than 0.1% recorded in the Clinical Practice Research Datalink as indeterminate gender. Most people (69.2%) were of white ethnicity, with 15.7% missing an ethnic group recording. Study participants were evenly distributed among the ten Index of Multiple Deprivation groups, but with relatively fewer (8.8%) in the most deprived tenth. Based on the single code definition (any code representing one of the 212 long term conditions present in the record), the number of long term conditions per person and prevalence of multimorbidity increased with age, and were higher in women and people of white ethnicity. Multimorbidity prevalence was highest in people living in the least deprived Index of Multiple Deprivation group (77.6%), with a declining prevalence up to the eighth group (70.0%) but with subsequently higher prevalence in the two most deprived groups (72.0% and 74.7%).

**Table 1:** Characteristics of study participants by mean number of conditions and prevalence of multimorbidity based on a single code definition of 212 long term conditions

Participant characteristics	Total, %	Mean number of long term conditions	Prevalence with one or more long term conditions, %	Prevalence with multimorbidity, %
Age (years):				
18-29	1 958 414 (20.2)	1.9	71.6	48.5
30-39	1 580 260 (16.3)	2.6	76.8	57.3
40-49	1 806 547 (18.6)	3.8	86.4	72.6
50-59	1 504 086 (15.5)	5.5	93.4	85.7
60-69	1 255 632 (12.9)	7.1	96.5	92.4
70-79	987 576 (10.2)	9.0	98.5	96.7
≥80	626 058 (6.4)	11.2	99.0	98.1
Gender:				
Female	4 831 012 (49.7)	5.3	89.4	78.5
Indeterminate	205 (<0.1)	4.2	88.3	74.1
Male	4 887 356 (50.3)	4.5	83.2	69.4
Ethnic group:				
White	6 721 471 (69.2)	5.5	90.2	79.6
South Asian	699 376 (7.2)	4.4	82.0	68.5
Black	390 621 (4.0)	4.0	83.8	68.8
Other	251 218 (2.6)	2.4	60.6	43.5
Mixed	132 957 (1.4)	3.4	79.7	62.8
Missing	1 522 930 (15.7)	3.3	76.4	58.6
Index of Multiple Deprivation group:				
1 (least deprived)	1 033 628 (10.6)	5.0	89.3	77.6
2	941 864 (9.7)	5.1	89.1	77.4
3	990 212 (10.2)	5.0	87.2	75.3
4	986 174 (10.1)	5.1	87.7	75.8
5	923 682 (9.5)	4.8	85.7	73.2
6	980 715 (10.1)	4.8	85.2	72.6
7	1 018 134 (10.5)	4.7	83.8	70.8
8	1 007 086 (10.4)	4.6	83.3	70.0
9	973 478 (10.0)	4.8	85.0	72.0
10 (most deprived)	855 807 (8.8)	5.2	86.8	74.7
Missing	7793 (0.1)	4.1	79.8	65.4
Total	9 718 573	4.9	86.3	73.9

### Multimorbidity prevalence

The prevalence of people with one or more, two or more, or three or more long term conditions varied substantially according to definition used (table 2). Under the single code definition, the prevalence of multimorbidity (two or more long term conditions) was 73.9%, reducing to 41.4% using the three codes within 12 months’ definition and 35.2% using only conditions recorded as active in the problem list.

**Table 2:** Prevalence of people with one or more, two or more, or three or more long term conditions using different definitions. Data are number (percentage)

Long term condition definition	One or more long term conditions	Two or more long term conditions	Three or more long term conditions
Single code	8 386 988 (86.3)	7 183 662 (73.9)	6 077 925 (62.5)
Two over three months	7 027 508 (72.3)	5 366 285 (55.2)	4 209 213 (42.3)
Two over 12 months	6 797 097 (69.9)	5 106 872 (52.5)	3 980 370 (41.0)
Three within 12 months	5 937 863 (61.1)	4 023 023 (41.4)	2 864 536 (29.5)
Any in past 12 months	5 964 391 (61.4)	4 149 619 (42.7)	3 041 805 (31.3)
Problems only	5 546 217 (57.1)	3 419 374 (35.2)	2 172 454 (22.4)

Of the 41 conditions included that were required to have multiple codes, elevated concentrations of total cholesterol, triglycerides, and low density lipoprotein; dermatitis; and enthesopathy and synovial disorders were the most common conditions, with a prevalence of at least 20% using the single code definition (online supplemental appendix figure A1). The prevalence of most of these conditions was substantially lower with each of the alternative definitions from two codes over three months to two codes over 12 months to three codes within 12 months, and any code in the past 12 months. However, reductions in the prevalence were not consistent between conditions, as evidenced by the prevalence of obesity, which was higher under the any in past 12 months definition compared with the three within 12 months definition.

For problem codes, the pattern was mixed according to condition. Hypertension, type 2 diabetes, thyroid disease, chronic kidney disease, atrial fibrillation, and chronic obstructive pulmonary disease had a similar prevalence applying the problems definition compared with using only the single code definition (online supplemental figure A2). However, those diagnoses based only on blood test results (raised concentrations of cholesterol or triglycerides) were represented rarely in the problem table (prevalence <0.1%; Online supplemental figure A2).

**Figure 3:**
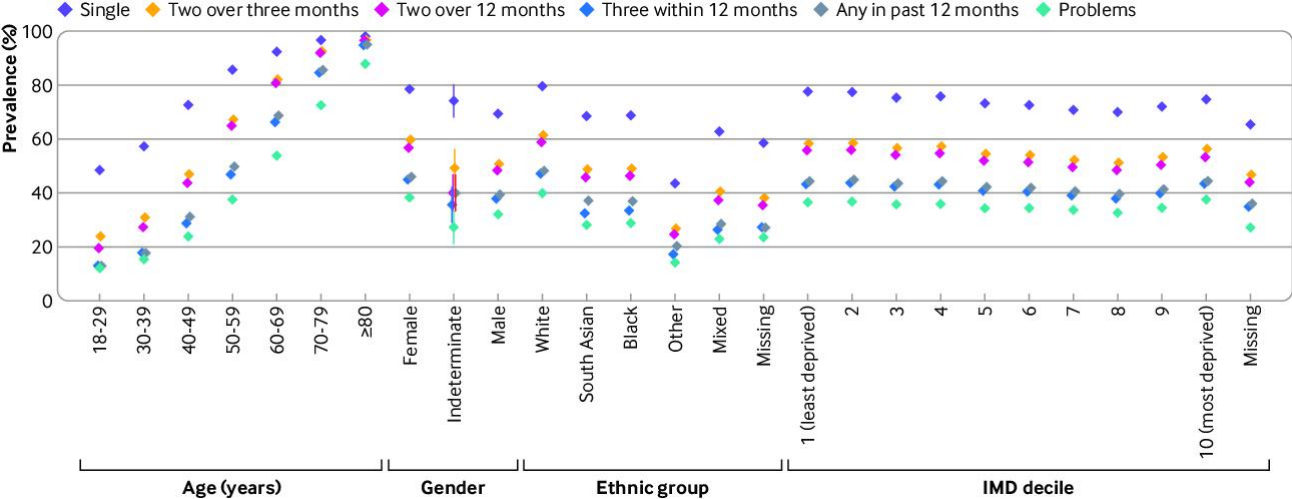
Prevalence of multimorbidity using different definitions of long term condition, by demographic factors. Diamonds represent estimates and bars represent 95% confidence intervals from mixed effects logistic regression models. IMD=Index of Multiple Deprivation

The prevalence of multimorbidity under each definition stratified by demographic group is shown in figure 3 (percentages are given in online supplemental table A2). Within all sociodemographic strata, multimorbidity prevalence was lowest applying the problems definition, from 12.1% in the 18-29 year age group to 87.8% in the 80 years and older age group, compared with 48.5% and 98.1%, respectively, applying a single code definition. Comparing the definitions using alternative timeframes, the absolute difference in prevalence between definitions was large for younger age groups but became smaller with increasing age. Within categories of gender, ethnic group, and Index of Multiple Deprivation group, absolute differences in multimorbidity prevalence between definitions remained similar in size (figure 3).

### Demographic differences in multimorbidity status according to disease timeframe

In people with multimorbidity based on the single code definition, we compared the expected probability of a change in multimorbidity status under each different timeframe, holding age, gender, ethnic group and Index of Multiple Deprivation at their mean values. The expected probabilities by definition of a person being reclassified as not multimorbid (compared with the single code definition) were 18.7% for two codes over three months, 21.9% for two codes over 12 months, 40.4% for three codes within 12 months, 37.5% for any code in the past 12 months, and 51.6% for problems.

**Figure 4:**
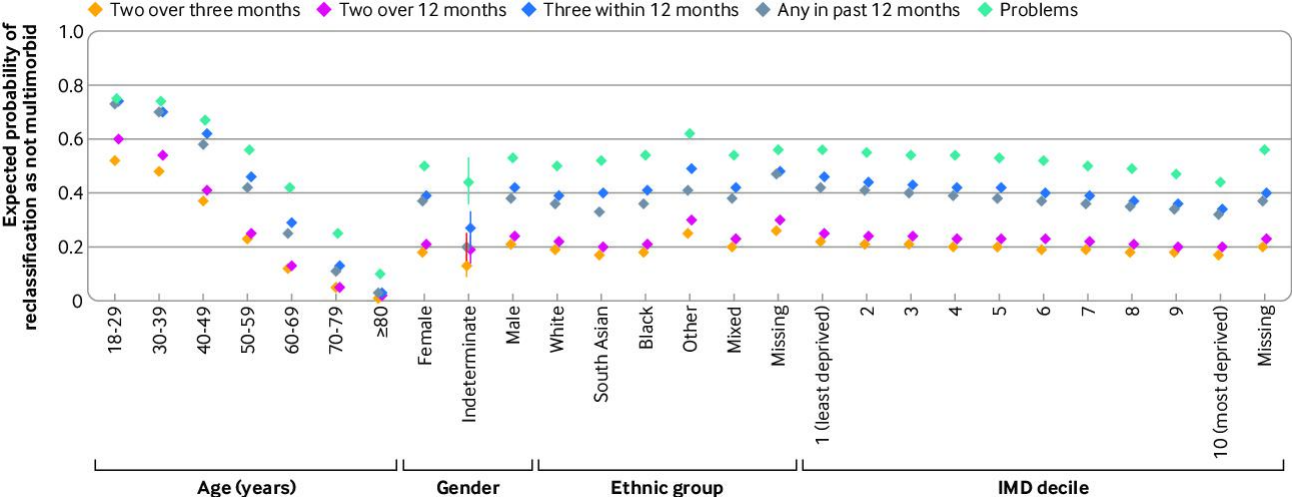
Expected probabilities for being reclassified as not multimorbid with each definition compared with a single code definition. Diamonds represent estimates and bars represent 95% confidence intervals from mixed effects logistic regression models

Older people were substantially less likely to be reclassified as not multimorbid compared with younger people after controlling for gender, ethnicity and deprivation, under each of the four alternative definitions compared with a single code definition (figure 4 and online supplemental table A3). People aged 80 years or older had only a 1-10% probability of being reclassified under the different definitions, compared with those aged 18-29 years, who had a 50-75% range of being reclassified. Within each definition, the expected probabilities of a change in multimorbidity status were similar for men and women. People of South Asian ethnicity had the lowest expected probability of a change in multimorbidity status, under all alternative definitions, except for the three codes within 12 months and problems definition, where the probability was lowest for people of white ethnicity. People of other, or unknown ethnic groups had the highest expected probabilities of no longer being multimorbid under all alternative definitions. The data showed a strong gradient towards a lower chance of being reclassified as not multimorbid with increasing deprivation across all alternative definitions.

### Demographic differences in the number of chronic conditions according to disease timeframe

We used mixed effects multivariable negative binomial models to estimate the expected reduction in the number of long term conditions under each definition compared with the single code definition, holding age, gender, ethnic group, and Index of Multiple Deprivation at their mean values. The mean expected reduction in the number of long term conditions from the single code definition was 1.9 for two codes over three months, 2.1 for two codes over 12 months, 3.1 for three codes within 12 months, 3.0 for any code in the past 12 months, and 3.6 for problems.

Younger people had a smaller expected reduction in the number of long term conditions than older people, after controlling for gender, ethnicity and deprivation, relative to the single code definition. This finding was apparent across all alternative definitions but was more pronounced in the problems, three within 12 months and any in past 12 months definitions, with people of 18-29 years expected to have 2.0 conditions fewer by each definition, compared with people 80 years or older expected to have 6.4, 3.9 and 4.0 conditions fewer, respectively, when compared with the single code definition (online supplemental appendix figure A3 and table A4). Men had smaller expected reductions in the count of conditions compared with women across all definitions. People of South Asian ethnicity had the largest expected reduction in the number of long term conditions, while people of black, other, or unknown ethnic groups had smaller reductions in the expected count. Data were trending towards larger expected reduction in counts of long term conditions in people living in areas of higher deprivation.

## Discussion

Our study found large variation in the prevalence of multimorbidity depending on the timeframe used to define long term conditions in a nationally representative cohort of patients in the general practice electronic health record in England, ranging from 41.4% where three codes were required within a 12 month period to 73.9% where a single code sufficed. Using active problem codes resulted in the lowest prevalence of 35.2%. A large variation was found despite using the same set of conditions and applying the alternative definitions for only 41 (19%) of the 212 conditions included. Prevalence of multimorbidity by sociodemographic factors varied substantially, particularly for age. Our selection of alternative timeframes represents a range of approaches, rather than a comprehensive assessment of all possibilities. Although we were unable to assess from the electronic health record whether people are appropriately reclassified using alternative timeframes, our results highlight the large disparities in estimates of multimorbidity prevalence, and that choice of timeframe will affect estimates for some people more than others.

Prevalence of multimorbidity reported in the existing literature ranges widely, with differences explained in part by the number of long term conditions included.^5^ We used a similar number of conditions to those of Head and colleagues who reported a prevalence of 52.8% in England in 2019.^3^ Their reported prevalence of multimorbidity was much higher than those from two other UK based studies of 23.2% and 27.2%, which used a smaller set of long term conditions (40 and 36, respectively, compared with Head et al's 211).^9 16^ Our most comparable definition (three codes within 12 months) resulted in a lower prevalence of multimorbidity (41.4%) than that of Head and colleagues, which may relate to our recategorisation of codes from the CALIBER study, and our different set of conditions that required multiple codes. Using a single code definition, the detected prevalence in our study was substantially higher (74%), likely reflecting inclusion of acute or inactive conditions. Our study is novel in identifying that the timeframe for defining long term conditions also has a substantial effect on prevalence of multimorbidity, comparable to the differences in the number of long term conditions on the prevalence of multimorbidity.

Our results also show that differences in prevalence estimates according to timeframe do not have uniform effects across a population. Large differences were found between demographic factors and the prevalence of multimorbidity and the number of long term conditions. Older age, South Asian ethnicity, and greater deprivation were associated with a lower risk of being reclassified as not multimorbid (figure 4) across all alternative definitions, but with corresponding larger reductions in the total count of diseases (online supplemental figure A3). Various sociodemographic factors are known to affect incidence and prevalence of multimorbidity, with previous studies highlighting that risk is higher in people living in areas of greater socioeconomic deprivation and people of black ethnicity.^3 17^ Although we found that those living in less deprived areas had a higher prevalence of multimorbidity when using a single code definition (table 1), this group were more likely to be reclassified as not multimorbid than people in more deprived groups when requiring multiple codes (figure 4). Our findings suggest differences in the composition of long term conditions contributing to multimorbidity in younger versus older age, and in people living in less versus more deprived areas. Groups that are more likely to be reclassified are more likely to have conditions where a single code could indicate an acute condition, for example, dermatitis and enthesopathy and synovial disorders, which were large contributors to overall multimorbidity burden (online supplemental figure A1). Other explanations for differences between groups might be explained by differential under-counting of disease burden or differences in health seeking behaviour and access to healthcare.^18 19^ Variation in how disease codes are entered between general practices and between clinicians within the same general practices which impact differently on patient groups.^20 21^

We hypothesised that using only conditions designated by clinicians as active problems in the healthcare record could be an effective route to identifying active long term conditions. This method yielded the lowest prevalence of multimorbidity of 35.2% overall, and of 87.8% in those aged 80 years and older, but with substantial variation between conditions. In primary care in England, the management of some long term conditions is incentivised by the quality and outcomes framework which started in 2004.^22^ We found that conditions such as hypertension, type 2 diabetes, and chronic obstructive pulmonary disease, all included in the framework, were much more similar in prevalence comparing the single code and problems definition. This suggests there may be coding bias towards conditions present in the framework when using problem codes, and so we do not recommend this method for studies of multimorbidity including a broad range of conditions.

### Strengths and limitations

A strength of our study is the use of large sample of adults registered to primary care in England, with previous studies of the Clinical Practice Research Datalink Aurum data finding it to be representative of the national population.^11^ We adopted a broad set of disease codes, many of which have particular relevance to primary care, and applied different definitions of long term condition timeframe using the same set of conditions to the same patient cohort. To an extent, these definitions are arbitrary, and our analysis presented a range of approaches used in the literature before, rather than attempting to analyse all possible definitions. For example, a condition lasting more than six months is sometimes used to define a long term condition,^6^ but we did not include this definition because estimates would lie between our range of three and 12 months.

A limitation of using routinely collected healthcare data is that some conditions may not be recorded, either because a person does not present with a condition or because when presenting, the condition is not coded by a clinician. The likelihood of missing codes is unlikely to be random with respect to either patients or diseases. Although a previous systematic review found good agreement in diseases recorded in Clinical Practice Research Datalink with other sources,^23^ comparison against cancer diagnoses in the Clinical Practice Research Datalink with cancer registry data found a range of 9–26% for different cancers were missing in the Clinical Practice Research Datalink.^24^ The financial incentives offered by quality and outcomes framework have improved data collection and coding in primary care^22^ and may therefore inflate the prevalence of long term conditions requiring multiple codes for conditions included in the framework relative to other conditions.

The code lists used for raised total cholesterol, raised low density lipoprotein cholesterol, low high density lipoprotein cholesterol and raised triglycerides available from the CALIBER study include test results (rather than diagnostic codes), which were rarely coded as problems, partially explaining the substantially lower prevalence of multimorbidity overall. Studies using problem codes alone would therefore need to include diagnostic codes, but was not feasible for comparison in our study as the granularity required in low density lipoprotein cholesterol and high density lipoprotein cholesterol measurements cannot be accounted for using diagnostic codes alone. A further issue of using problem codes is in defining active problems. In Clinical Practice Research Datalink Aurum no date is recorded at which a problem is changed from active to inactive. Our data extraction occurred in May 2022, and so some codes marked as inactive were likely active at the study start date, and so our findings may represent an underestimate. However, given our focus on chronic conditions, the number of conditions to have resolved is unlikely to be large.

We used a large set of chronic conditions, which will have unequal burden on clinical outcomes, healthcare use, and quality of life. Many may not be included in other multimorbidity measures, with a recent Delphi study identifying a core set of 24 conditions to always include and 35 to usually include and with no conditions identified for exclusion.^25^ A smaller and standardised set of conditions can aid comparability and reproducibility but has drawbacks. Firstly, a smaller set limits the scope to detect novel associations between less common conditions. Secondly, choices over conditions are subjective and those that are less frequent can still have a large burden on individuals.

The overlap between some of the long term conditions included here may lead to double counting and over-estimation of multimorbidity, for example, combining myocardial infarction and angina as ischaemic heart disease. However, when investigating the cause of disease, more granular categories may be a benefit because a person with a myocardial infarction who subsequently develops angina may have a different trajectory and opportunities for intervention and prevention to a person with angina who subsequently develops a myocardial infarction, despite a common pathophysiology.

Combining diagnostic codes with relevant medications or treatments may help to distinguish active versus inactive conditions; for example, use of a proton pump inhibitor in gastritis. Similarly, the quality and outcomes framework excludes patients from the asthma register if no asthma related drugs were prescribed in the past 12 months.^26^ However, a difficulty with this approach for some diseases is that drugs can have multiple indications; for example, proton pump inhibitors being co-prescribed with non-steroidal anti-inflammatory medications, which may be more common in those with a history of gastro-intestinal problems irrespective of active symptoms.^27^

### Implications

Despite the consensus in the medical literature on the definition of multimorbidity as the co-occurrence of two or more chronic conditions,^4^ which conditions should be included or how to determine chronicity has not been agreed.^5 25^ Our findings highlight that even when using the same set of medical codes, decisions on how to define a long term condition can change the prevalence of multimorbidity almost twofold, which has important implications for the direct comparison of estimates between studies. Results also suggest that a universally agreed metric of multimorbidity prevalence may be an unrealistic target and that estimates are highly context dependent. Rather than seeking one rigid definition of multimorbidity, we believe that researchers should instead embrace the variety of approaches, better reflecting the variety of lived experiences of people having multimorbidity. As such, we do not advocate only one of our approaches as the best, with choice dependent in part on the aims of the research. Nevertheless, some approaches may be more suitable in particular contexts, while the impact of bias, and exclusion of specific groups from multimorbidity measures, highlighted in our work, should be assessed (Box 1).

Box 1 **Recommendations for research in applying a timeframe for counting long term conditions in the electronic health record**

1. Provide a rationale for the choice of timeframe to define a long term condition
2. For studies of disease causes, use of a single code anywhere in the record may be preferred, as a condition even if historic and inactive may be relevant to subsequent disease development
3. Analyses of current interactions with healthcare services may be best suited to using codes appearing in a recent timeframe, for example, within the past 12 months
4. Timeframes requiring more than one code may be biased by factors related to repeated coding, therefore, we recommend considering sensitivity analyses using only a single code definition

Researchers should consider which conditions to include, and may opt for a narrower set of diseases by inclusion of only conditions for which a single code would indicate chronic risk, rather than those that may also represent acute conditions. Where inclusion of a greater breadth of diseases is preferred, researchers should decide whether some of these require presence of multiple codes over time. For studies focused on cause or accumulation of diseases over time, use of any diagnostic code in the record may be the preferred approach, as a disease, even if not currently active and only recorded once, may be relevant to subsequent disease development. However, this approach may lead to inclusion of acute or inactive conditions, inflating the prevalence of multimorbidity. For studies focused on associations between current disease burden and healthcare service use and treatment, use of more contemporaneous active diseases may be preferable. For example, use of codes from the past 12 months, or by incorporating prescription data where possible for some conditions. However, any approach that uses multiple codes may be at greater risk of bias in the frequency of coding of conditions, which may depend on factors related to the patient, clinician, general practice, and coding incentives.^21^ Therefore, we suggest that use of a single code definition should always be considered as a sensitivity analysis to understand the effect on prevalence and differential impact between patients.

## Conclusion

The choice of timeframe to define the chronicity of a long term condition based on code frequency in the electronic health record has a substantial effect on the reported prevalence of multimorbidity. Younger people, people of some ethnic groups, and people living in areas of lower socioeconomic deprivation were more likely to be reclassified as not multimorbid under alternative timeframes compared with using a single code, highlighting the need to consider the potential bias arising from the choice of definition.

## Data Availability

Data may be obtained from a third party and are not publicly available. The data used in this study are not publicly available as access is subject to approval processes. More information is available from Clinical Practice Research Datalink: https://cprd.com/research-applications.
