# Supplementary material for: Effect of timeframes to define long term conditions and sociodemographic factors on prevalence of multimorbidity using disease code frequency in primary care electronic health records: retrospective study
Source: BMJ Med. 2024 Feb 13;3(1):e000474. doi: 10.1136/bmjmed-2022-000474 (PMC10868275; doi:10.1136/bmjmed-2022-000474)
Supplement: Supplementary data [file bmjmed-2022-000474supp001.pdf]

Appendix

Impact and inequalities in the prevalence of multimorbidity using different timeframes to define long term conditions: retrospective study of disease code frequency in primary care electronic healthcare records

Beaney T[1,2] (0000-0001-9709-7264), Clarke J[2], Woodcock T[1], Majeed A[1], Barahona M[2], Aylin P[1]

1. Department of Primary Care and Public Health, Imperial College London, London, W6 8RP, United Kingdom
2. Centre for Mathematics of Precision Healthcare, Department of Mathematics, Imperial College London, London, SW7 2AZ, United Kingdom

Corresponding Author:
Dr Thomas Beaney
Department of Primary Care and Public Health, Imperial College London, London, W6 8RP, United Kingdom
Email: thomas.beaney@imperial.ac.uk

This section summarises the data cleaning approach. Code lists for ethnicity and disease code lists are available from our GitHub repository: https://tbeaney.github.io/MMclustering/

Observation data is included in the form of Medcodes. We included observations only where the date of observation and the date of data entry occurred on or before 1st January 2020. Duplicate observations were excluded.

Ethnicity

We used ethnicity codes developed by Davidson and colleagues.[1] We removed four codes from these list representing examination findings, as suggesting clinician observed findings rather than reported by the patient. We followed the algorithm from Mathur *et al* to identify ethnicity:[2]

1. Remove duplicate ethnicity codes recorded on the same date
2. Assign each code to the 5 and 16 level ethnicity categories
3. If only one ethnicity code, use single code to define ethnicity
4. If more than one code, use most frequently occurring ethnicity code
5. If more than one code with the same count, use the latest ethnicity code instead.

Chronic conditions

These were mapped using code lists developed for the CALIBER study, and adapted for use in multimorbidity in CPRD Aurum.[3,4] We reviewed the codes in these lists, and made amendments to the code lists for diabetes, to remove Type 1 and Type 2 codes from the other/unspecified code list. We added chronic primary pain to the set of included conditions and created a new code list. Previous studies of multimorbidity in primary care settings have found a high prevalence and burden of chronic pain.[5,6] However, in order to avoid double counting of pain related to another chronic condition included, we excluded secondary causes, and included only primary pain conditions. Codes were reviewed by two clinicians and co-authors, TB and JC, and conditions requiring multiple codes were selected based on the following criteria:

1. A disease category indicates symptoms, e.g. urinary incontinence/dysmenorrhoea.
2. A disease category indicates a disease which may be acute or present as a single short-lived episode, e.g. gastritis, pancreatitis, sinusitis, depression, anxiety.
3. A disease category may be easily confused with another disease and be diagnosed in primary care clinically without investigations, e.g. skin disorders, asthma.
4. A disease category may indicate the result of a single blood test, e.g. anaemias (except for aplastic anaemia). The disease code of Chronic Kidney Disease (CKD) is not included here as the definition implies two tests at least 3 months apart.

Where these criteria were not met, then a single code was deemed sufficient for diagnosis:

1. Abdominal Aortic Aneurysm
2. Abdominal Hernia
3. Alcoholic liver disease
4. Angiodysplasia of colon
5. Ankylosing spondylitis
6. Aplastic anaemias
7. Asbestosis
8. Atrial Fibrillation
9. Atrioventricular blocks
10. Autism and Asperger's syndrome
11. Autoimmune liver disease
12. Autonomic Neuropathy
13. Barrett's oesophagus
14. Benign Prostatic Hyperplasia
15. Visual impairment and blindness
16. Bronchiectasis
17. Cardiomyopathy: other
18. Cataract
19. Cerebral Palsy
20. Chronic Fatigue Syndrome
21. Chronic Kidney Disease
22. COPD
23. Chronic primary pain
24. Chronic viral hepatitis
25. Cirrhosis
26. Coeliac disease
27. Collapsed vertebra
28. Congenital Septal Defect
29. Coronary Heart Disease (not otherwise specified)
30. Crohn's disease
31. Cystic Fibrosis
32. Hearing loss
33. Dementia
34. Diabetes Mellitus: other or not specified
35. Diabetic Eye Disease
36. Diabetic Neuropathy
37. Diaphragmatic hernia
38. Dilated cardiomyopathy
39. Diverticular Disease
40. Down's syndrome
41. Eating Disorders
42. Endometrial hyperplasia and hypertrophy
43. Endometriosis

44. Enteropathic arthropathy
45. Epilepsy
46. Benign essential tremor
47. Fatty Liver
48. Fibromatosis
49. Giant Cell arteritis
50. Glaucoma
51. Glomerulonephritis
52. Gout
53. Heart failure
54. HIV
55. Hodgkin Lymphoma
56. Hyperkinetic disorders
57. Hyperparathyroidism
58. Hypersplenism
59. Hypertension
60. Hypertrophic Cardiomyopathy
61. Hyposplenism
62. Idiopathic Intracranial Hypertension
63. Immunodeficiencies
64. Intellectual disability
65. Intervertebral disc disorders
66. Intracerebral haemorrhage
67. Ischaemic stroke
68. Left bundle branch block
69. Leukaemia
70. Hepatic failure
71. Lupus Erythematosus
72. Macular degeneration
73. Meniere's Disease
74. Motor neurone disease
75. Multiple sclerosis
76. Multiple valve disorder
77. Myasthenia gravis
78. Myelodysplastic Syndrome
79. Myocardial Infarction
80. Neuropathic Bladder
81. Non-Hodgkin Lymphoma
82. Nonrheumatic aortic valve disorders
83. Nonrheumatic mitral valve disorders
84. Obsessive-compulsive disorder
85. Obstructive and reflux uropathy
86. Oesophageal varices
87. Osteoarthritis (excl spine)
88. Osteoporosis
89. Other haemolytic anaemias
90. Parkinson's disease
91. Peripheral Arterial Disease
92. Peripheral Neuropathy
93. Personality disorders
94. Plasma Cell Malignancy
95. Pleural plaque
96. Polycystic ovarian syndrome
97. Polycythaemia vera
98. Polymyalgia Rheumatica
99. Portal hypertension
100. Primary Malignancy: Biliary Tract
101. Primary Malignancy: Bladder
102. Primary Malignancy: Bone
103. Primary Malignancy: Bowel
104. Primary Malignancy: Brain

105. Primary Malignancy: Breast
106. Primary Malignancy: Cervix
107. Primary Malignancy: Kidney
108. Primary Malignancy: Liver
109. Primary Malignancy: Lung
110. Primary Malignancy: Melanoma
111. Primary Malignancy: Mesothelioma
112. Primary Malignancy: Multiple Sites
113. Primary Malignancy: Oesophageal
114. Primary Malignancy: Oropharyngeal
115. Primary Malignancy: other
116. Primary Malignancy: Ovary
117. Primary Malignancy: Pancreas
118. Primary Malignancy: Prostate
119. Primary Malignancy: Skin
120. Primary Malignancy: Stomach
121. Primary Malignancy: Testis
122. Primary Malignancy: Thyroid
123. Primary Malignancy: Uterus
124. Primary pulmonary hypertension
125. Primary Thrombocytopaenia
126. Psoriatic Arthritis
127. Pulmonary Fibrosis
128. Raynaud's syndrome
129. Retinal vascular occlusions
130. Rheumatic Valve Disorder
131. Rheumatoid Arthritis
132. Right bundle branch block combinations
133. Sarcoidosis
134. Schizophrenia
135. Scleritis and episcleritis
136. Scleroderma
137. Secondary Malignancy: Adrenal Gland
138. Secondary Malignancy: Bone
139. Secondary Malignancy: Bowel
140. Secondary Malignancy: Brain
141. Secondary Malignancy: Liver
142. Secondary Malignancy: Lung
143. Secondary Malignancy: Lymph Nodes
144. Secondary Malignancy: other
145. Secondary Malignancy: Peritoneum
146. Secondary Malignancy: Pleura

147. Secondary polycythaemia
148. Secondary pulmonary hypertension
149. Secondary Thrombocytopaenia
150. Sick sinus syndrome
151. Sickle Cell Disease
152. Sjogren's Syndrome
153. Spina bifida
154. Spinal stenosis
155. Spondylolisthesis
156. Spondylosis
157. Stable Angina
158. Stroke: not otherwise specified
159. Subarachnoid haemorrhage
160. Supraventricular tachycardia
161. Thalassaemia
162. Thrombophilia
163. Thyroid Disease
164. Transient ischaemic attack
165. Tubulo-interstitial nephritis
166. Type 1 Diabetes Mellitus
167. Type 2 Diabetes Mellitus
168. Ulcerative colitis
169. Unstable Angina
170. Venous thromboembolic disease (Excl PE)
171. Ventricular tachycardia

The following 41 diseases required multiple codes:

1. Acne
2. Alcohol Misuse
3. Allergic and chronic rhinitis
4. Alopecia areata
5. Anaemia: other
6. Anterior and Intermediate Uveitis
7. Anxiety disorders
8. Asthma
9. Bipolar affective disorder and mania
10. Chronic Cystitis
11. Chronic sinusitis
12. Depression
13. Dermatitis
14. Dysmenorrhoea
15. Enthesopathy and synovial disorder
16. Folate deficiency anaemia
17. Gastritis and duodenitis
18. Gastro-oesophageal reflux disease
19. Hidradenitis suppurativa
20. Hypertrophic Nasal Turbinates
21. Irritable bowel syndrome
22. Low HDL-C
23. Migraine
24. Obesity
25. Pancreatitis
26. Pericardial Effusion
27. Pleural effusion
28. Posterior Uveitis
29. Psoriasis
30. Raised LDL-C
31. Raised Total Cholesterol

32. Raised Triglycerides
33. Rosacea
34. Seborrheic dermatitis
35. Sleep apnoea
36. Substance Misuse
37. Tinnitus
38. Trigeminal neuralgia
39. Urinary Incontinence
40. Vitamin B12 deficiency anaemia
41. Vitiligo

Data were de-duplicated based on disease categories on the same date, i.e., where two different codes indicating the same disease were recorded on the same day, only one was counted.

For diabetes, many codes are used for both Type 1 and Type 2 diabetes, e.g., ‘Diabetes mellitus’ and ‘O/E – diabetic maculopathy present both eyes’. Furthermore, the initial diagnosis may be mis-classified, with the correct diabetes type later recorded. To avoid double or triple counting multimorbidity burden in people with diabetes, we selected the most likely diabetes type using the following algorithm:

1. Count all occurrences of Type 1, Type 2 and unspecified/other codes for each patient
2. If only one type, then assign this as disease type
3. If Type 1 and no Type 2 codes, then assign as Type 1
4. If Type 2 and no Type 1 codes, then assign as Type 2
5. If a mix of Type 1 and Type 2 codes, then look within last 3 years of observations:
    a. If Type 1 and no Type 2 codes, then assign as Type 1
    b. If Type 2 and no Type 1 codes, then assign as Type 2
    c. If a mix of both Type 1 and Type 2 codes, then assign as unspecified

Four diseases in the CALIBER code lists were defined by blood tests alone (Raised Total Cholesterol, Raised LDL-C, Low HDL-C and Raised Triglycerides). We retained only values with abnormal readings defined in the CALIBER study and made distributional assumptions regarding the units (mmol/L or mg/dL) where the defined units were implausible (Table A1). In the original CALIBER study, CKD also included blood test results, based on the eGFR, but we did not include those here, and included CKD only where a diagnostic code was present.

Table A1: mapping of cholesterol and triglyceride measurements

disease	gender	measurement	Operator1	Value1	Operator2	Value2	units
Raised Total Cholesterol		Serum	>	5	<	38	mmol/L
Raised Total Cholesterol		Serum	>	193.35			mg/dL
Raised Total Cholesterol		Plasma	>	4.8544	<	36	mmol/L
Raised Total Cholesterol		Plasma	>	187.7184			mg/dL
Raised LDL-C		Serum	>	3	<	38	mmol/L
Raised LDL-C		Serum	>	116.01			mg/dL
Raised LDL-C		Plasma	>	2.9126	<	36	mmol/L
Raised LDL-C		Plasma	>	112.6311			mg/dL
Low HDL-C	F	Serum	<	1.2	>	12	mmol/L
Low HDL-C	F	Serum	<	46.404			mg/dL
Low HDL-C	F	Plasma	<	1.165	>	12	mmol/L
Low HDL-C	F	Plasma	<	45.0524			mg/dL
Low HDL-C	M	Serum	<	1	>	10	mmol/L
Low HDL-C	M	Serum	<	38.67			mg/dL
Low HDL-C	M	Plasma	<	0.9709	>	10	mmol/L
Low HDL-C	M	Plasma	<	37.5437			mg/dL
Low HDL-C	I	Serum	<	1	>	10	mmol/L
Low HDL-C	I	Serum	<	38.67			mg/dL
Low HDL-C	I	Plasma	<	0.9709	>	10	mmol/L
Low HDL-C	I	Plasma	<	37.5437			mg/dL
Raised Triglycerides		Serum	>	1.7		38	mmol/L
Raised Triglycerides		Serum	>	150.569			mg/dL
Raised Triglycerides		Plasma	>	1.6521		36	mmol/L
Raised Triglycerides		Plasma	>	146.3256			mg/dL

Adapted from Kuan *et al* (2019)[4]; F = female, M = male, I = indeterminate

Body Mass Index (BMI) incorporated both codes and BMI measurements. We included only BMI measurements taken from age 16 years and adapted the approach used by Bhaskaran and colleagues with CPRD data:[7]

1. Weight records outside the range 20kg – 450 kg dropped.
2. Height records outside the range 121 – 214 cm dropped.
3. Duplicated records dropped.
4. BMI calculated as weight/height2 for all those with height and weight on the same day

5. Where a weight was recorded on a given date, but no height recording, the most recent older height record was used.
6. Remaining weight records without a matching height, the first available future height measurement was used.
7. Where BMI could not be calculated on a given date with a height or weight measurement, but a BMI was recorded in CPRD, this was used in preference.
8. BMIs outside the range 5 – 200 kg were dropped.

Problem definition

In CPRD, a separate ‘Problem’ table records observation codes that have been marked as problems. All entries in the Problem table link to one (and only one) corresponding observation in the Observation table. We extracted all active problems in the Problem table that had a corresponding link to a code in the Observation table, using the *obsid* variable. 12.1% of our extracted observation codes had a link to a problem code.

Statistical analysis

1. Linear equation for the mixed effects logistic regression model:

$$logit(y_{i,j}) = \beta_0 + \beta_1 age_{i,j} + \beta_2 gender_{i,j} + \beta_3 ethnicity_{i,j} + \beta_4 IMD_{i.j} + u_j$$

Where *j* represents the GP practice and *i* represents each patient in practice *j* and:

$$y_{i,j} = \begin{cases} 0, & x_{single,i,j} = x_{alternative,i,j} \\ 1, & x_{single,i,j} \neq x_{alternative,i,j} \end{cases}$$

And where $x_{single,i,j}$ and $x_{alternative,i,j}$ are binary indicators for having multimorbidity under the single code definition and each of the alternative definitions, respectively, in patient *i* in practice *j*.

2. Linear equation for the mixed effects negative binomial regression model:

$$log(y_{i,j}) = \beta_0 + \beta_1 age_{i,j} + \beta_2 gender_{i,j} + \beta_3 ethnicity_{i,j} + \beta_4 IMD_{i.j} + u_j$$

Where *j* represents the GP practice and *i* represents each patient in practice *j* and $y_{i,j}$ is the change in the number of LTCs for patient *i* in GP practice *j* for each definition, compared to a single code definition.

Table A2: prevalence of multimorbidity by demographic group according to each definition of chronic disease

Variable	Single	2 over 3	2 over 12	3 within 12	Any in last 12	Problems
**Age (years)**						
18-29	48.5%	23.9%	19.6%	13.1%	13.0%	12.1%
30-39	57.3%	30.9%	27.3%	17.8%	17.8%	15.4%
40-49	72.6%	46.9%	43.7%	28.7%	31.2%	23.9%
50-59	85.7%	67.2%	64.9%	46.9%	49.8%	37.5%
60-69	92.4%	82.1%	80.7%	66.3%	68.7%	53.8%
70-79	96.7%	92.5%	91.9%	84.6%	85.6%	72.5%
80+	98.1%	96.7%	96.6%	94.8%	95.0%	87.8%
**Gender**						
Female	78.5%	59.8%	56.8%	45.0%	46.0%	38.3%
Indeterminate	74.1%	49.3%	40.0%	35.6%	40.0%	27.3%
Male	69.4%	50.7%	48.4%	37.9%	39.4%	32.1%
**Ethnicity**						
White	79.6%	61.5%	58.8%	47.2%	48.2%	39.9%
South Asian	68.5%	48.8%	45.8%	32.5%	37.1%	28.2%
Black	68.8%	49.0%	46.3%	33.5%	36.9%	28.8%
Other	43.5%	26.8%	24.7%	17.3%	20.3%	14.2%
Mixed	62.8%	40.6%	37.3%	26.4%	28.5%	22.9%
Missing	58.6%	38.2%	35.5%	27.3%	27.2%	23.6%
**IMD decile**						
1 (least deprived)	77.6%	58.3%	55.8%	43.3%	44.3%	36.5%
2	77.4%	58.5%	56.0%	43.7%	44.9%	36.8%
3	75.3%	56.7%	54.1%	42.4%	43.6%	35.8%
4	75.8%	57.3%	54.7%	43.2%	44.4%	35.9%
5	73.2%	54.6%	52.0%	40.8%	42.2%	34.4%
6	72.6%	54.1%	51.4%	40.5%	41.9%	34.4%
7	70.8%	52.2%	49.6%	39.1%	40.6%	33.7%
8	70.0%	51.2%	48.5%	38.0%	39.7%	32.6%
9	72.0%	53.3%	50.4%	39.9%	41.3%	34.5%
10 (most deprived)	74.7%	56.4%	53.3%	43.5%	44.4%	37.6%
Missing	65.4%	46.8%	44.0%	34.9%	36.1%	27.2%

Figure A1: Prevalence of conditions requiring multiple codes under alternative timeframes for diagnosis (for conditions with at least 2% prevalence)

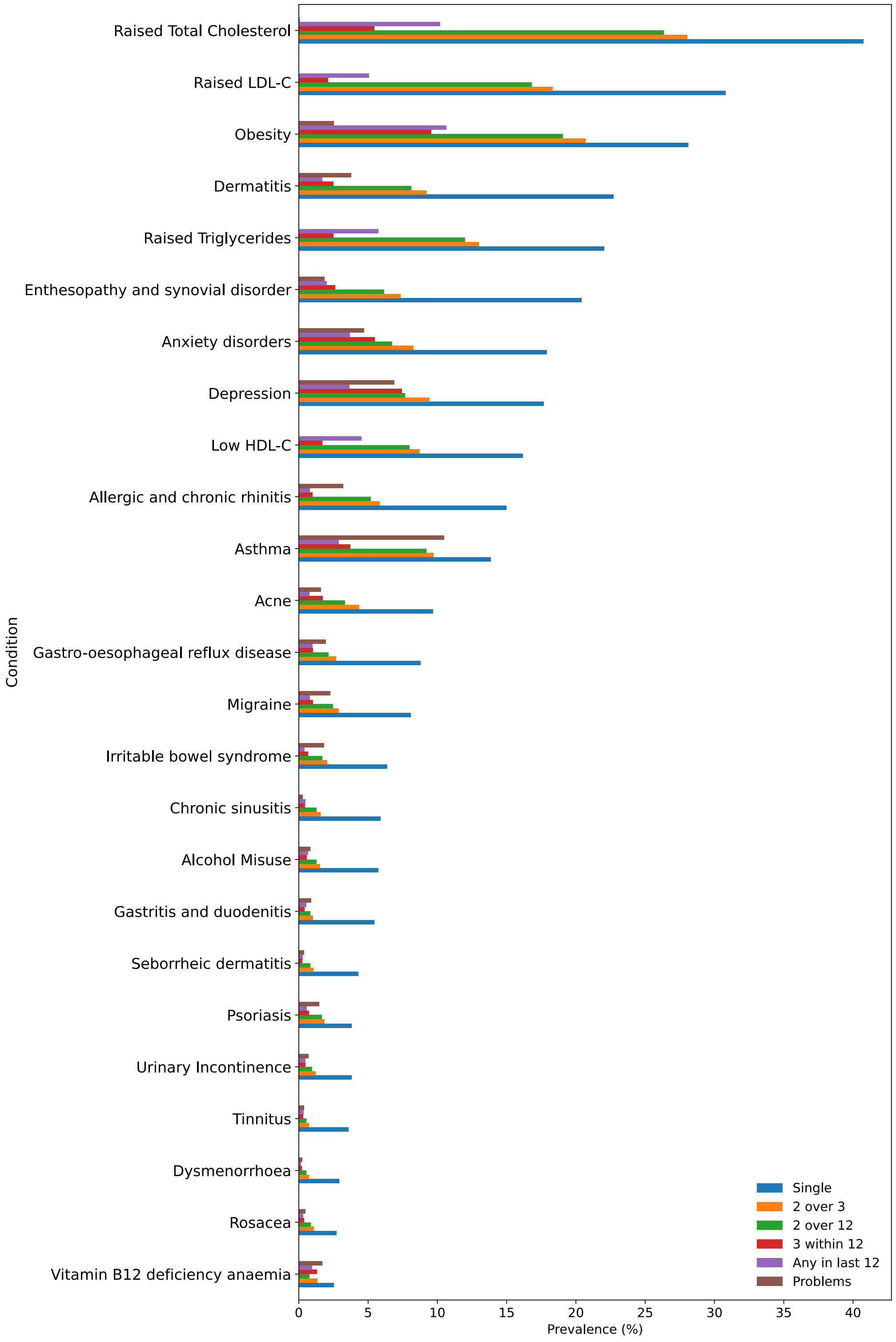

Figure A2: Prevalence of conditions using codes appearing in the problem table compared to a single code definition (for conditions with at least 1% prevalence)

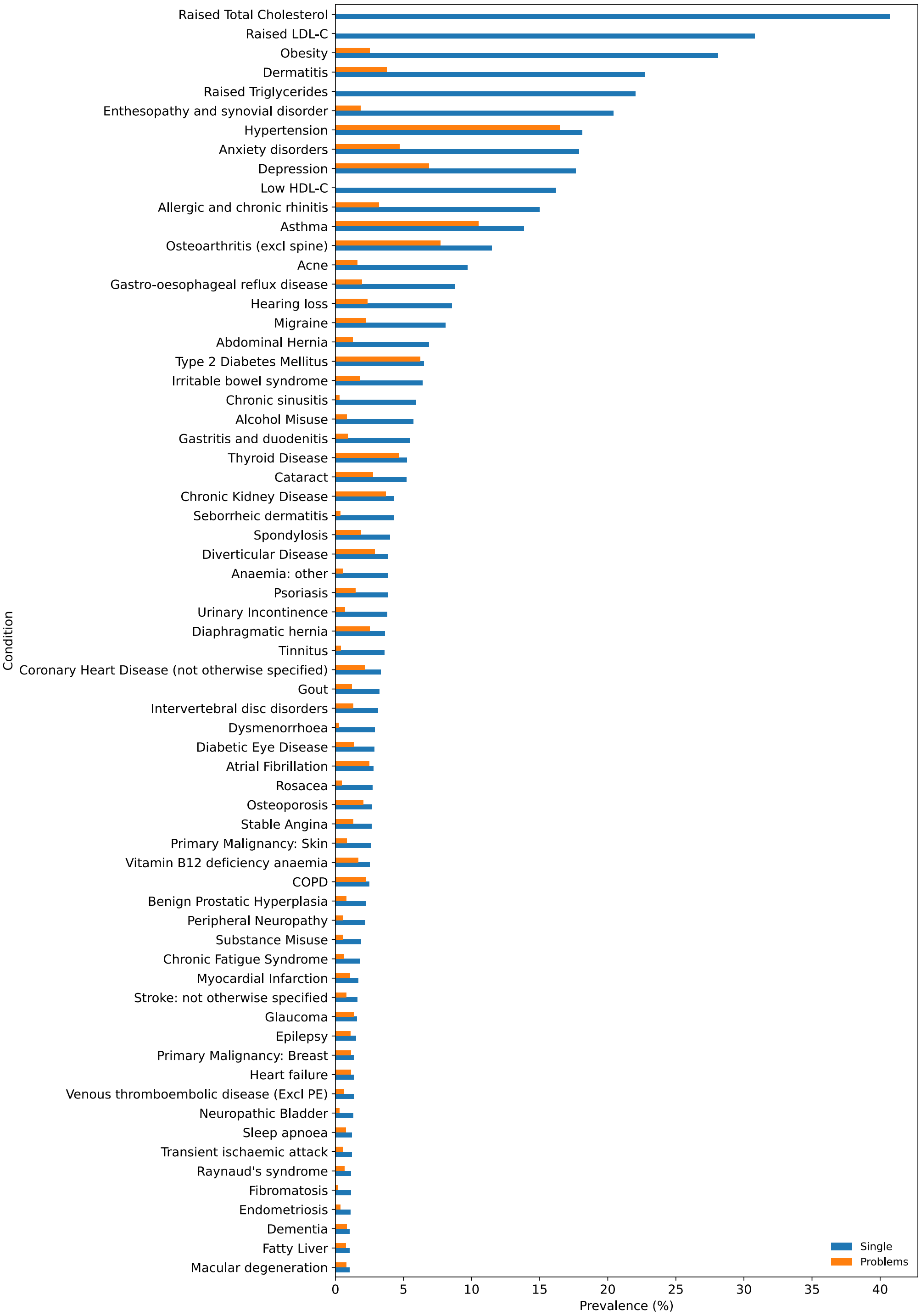

Table A3: Expected probabilities and 95% confidence intervals for being reclassified as not multimorbid with each definition compared to a single code definition

VARIABLE	DEFINITION: 2 over 3		2 over 12		3 within 12		Any in last 12		Problems	
	Coefficient	95% CI	Coefficient	95% CI	Coefficient	95% CI	Coefficient	95% CI	Coefficient	95% CI
**AGE CATEGORY (YEARS)**										
18-29	0.52	0.51 - 0.52	0.60	0.60 - 0.61	0.74	0.74 - 0.75	0.73	0.73 - 0.74	0.75	0.75 - 0.75
30-39	0.48	0.48 - 0.48	0.54	0.54 - 0.54	0.70	0.70 - 0.71	0.70	0.70 - 0.70	0.74	0.74 - 0.74
40-49	0.37	0.37 - 0.37	0.41	0.41 - 0.41	0.62	0.61 - 0.62	0.58	0.57 - 0.58	0.67	0.67 - 0.67
50-59	0.23	0.22 - 0.23	0.25	0.25 - 0.25	0.46	0.46 - 0.47	0.42	0.42 - 0.42	0.56	0.56 - 0.56
60-69	0.12	0.12 - 0.12	0.13	0.13 - 0.13	0.29	0.28 - 0.29	0.25	0.25 - 0.26	0.42	0.41 - 0.42
70-79	0.05	0.04 - 0.05	0.05	0.05 - 0.05	0.13	0.12 - 0.13	0.11	0.11 - 0.11	0.25	0.25 - 0.25
80 or more	0.01	0.01 - 0.01	0.02	0.02 - 0.02	0.03	0.03 - 0.03	0.03	0.03 - 0.03	0.10	0.10 - 0.10
**GENDER**										
Female	0.18	0.18 - 0.18	0.21	0.21 - 0.22	0.39	0.39 - 0.39	0.37	0.36 - 0.37	0.50	0.50 - 0.50
Indeterminate	0.13	0.09 - 0.17	0.19	0.14 - 0.24	0.27	0.20 - 0.33	0.20	0.15 - 0.25	0.44	0.36 - 0.53
Male	0.21	0.21 - 0.21	0.24	0.24 - 0.24	0.42	0.42 - 0.42	0.38	0.38 - 0.39	0.53	0.53 - 0.53
**ETHNICITY CATEGORY**										
White	0.19	0.19 - 0.19	0.22	0.21 - 0.22	0.39	0.39 - 0.39	0.36	0.36 - 0.37	0.50	0.50 - 0.50
South Asian	0.17	0.17 - 0.18	0.20	0.20 - 0.21	0.40	0.40 - 0.40	0.33	0.33 - 0.33	0.52	0.52 - 0.52
Black	0.18	0.18 - 0.19	0.21	0.21 - 0.21	0.41	0.40 - 0.41	0.36	0.35 - 0.36	0.54	0.54 - 0.54
Other	0.25	0.25 - 0.26	0.30	0.29 - 0.30	0.49	0.49 - 0.50	0.41	0.41 - 0.42	0.62	0.62 - 0.62
Mixed	0.20	0.20 - 0.20	0.23	0.23 - 0.24	0.42	0.42 - 0.43	0.38	0.38 - 0.38	0.54	0.54 - 0.54
Unknown	0.26	0.25 - 0.26	0.30	0.29 - 0.30	0.48	0.47 - 0.48	0.47	0.47 - 0.47	0.56	0.56 - 0.56
**IMD DECILE**										
1 (least deprived)	0.22	0.21 - 0.22	0.25	0.25 - 0.25	0.46	0.45 - 0.46	0.42	0.42 - 0.43	0.56	0.56 - 0.56
2	0.21	0.21 - 0.21	0.24	0.24 - 0.24	0.44	0.44 - 0.44	0.41	0.40 - 0.41	0.55	0.55 - 0.55
3	0.21	0.21 - 0.21	0.24	0.24 - 0.24	0.43	0.43 - 0.44	0.40	0.40 - 0.40	0.54	0.54 - 0.54
4	0.20	0.20 - 0.21	0.23	0.23 - 0.24	0.42	0.42 - 0.43	0.39	0.39 - 0.40	0.54	0.54 - 0.54
5	0.20	0.20 - 0.20	0.23	0.23 - 0.23	0.42	0.41 - 0.42	0.38	0.38 - 0.39	0.53	0.53 - 0.53
6	0.19	0.19 - 0.20	0.23	0.22 - 0.23	0.40	0.40 - 0.41	0.37	0.37 - 0.38	0.52	0.52 - 0.52
7	0.19	0.19 - 0.19	0.22	0.22 - 0.22	0.39	0.39 - 0.39	0.36	0.36 - 0.37	0.50	0.50 - 0.50
8	0.18	0.18 - 0.18	0.21	0.21 - 0.21	0.37	0.37 - 0.38	0.35	0.35 - 0.35	0.49	0.49 - 0.49
9	0.18	0.17 - 0.18	0.20	0.20 - 0.21	0.36	0.35 - 0.36	0.34	0.33 - 0.34	0.47	0.47 - 0.47
10 (most deprived)	0.17	0.17 - 0.17	0.20	0.19 - 0.20	0.34	0.33 - 0.34	0.32	0.31 - 0.32	0.44	0.43 - 0.44
Missing	0.20	0.19 - 0.21	0.23	0.22 - 0.24	0.40	0.38 - 0.42	0.37	0.35 - 0.38	0.56	0.54 - 0.57

Figure A3: Expected reduction in number of long-term conditions with each definition, compared to a single code definition

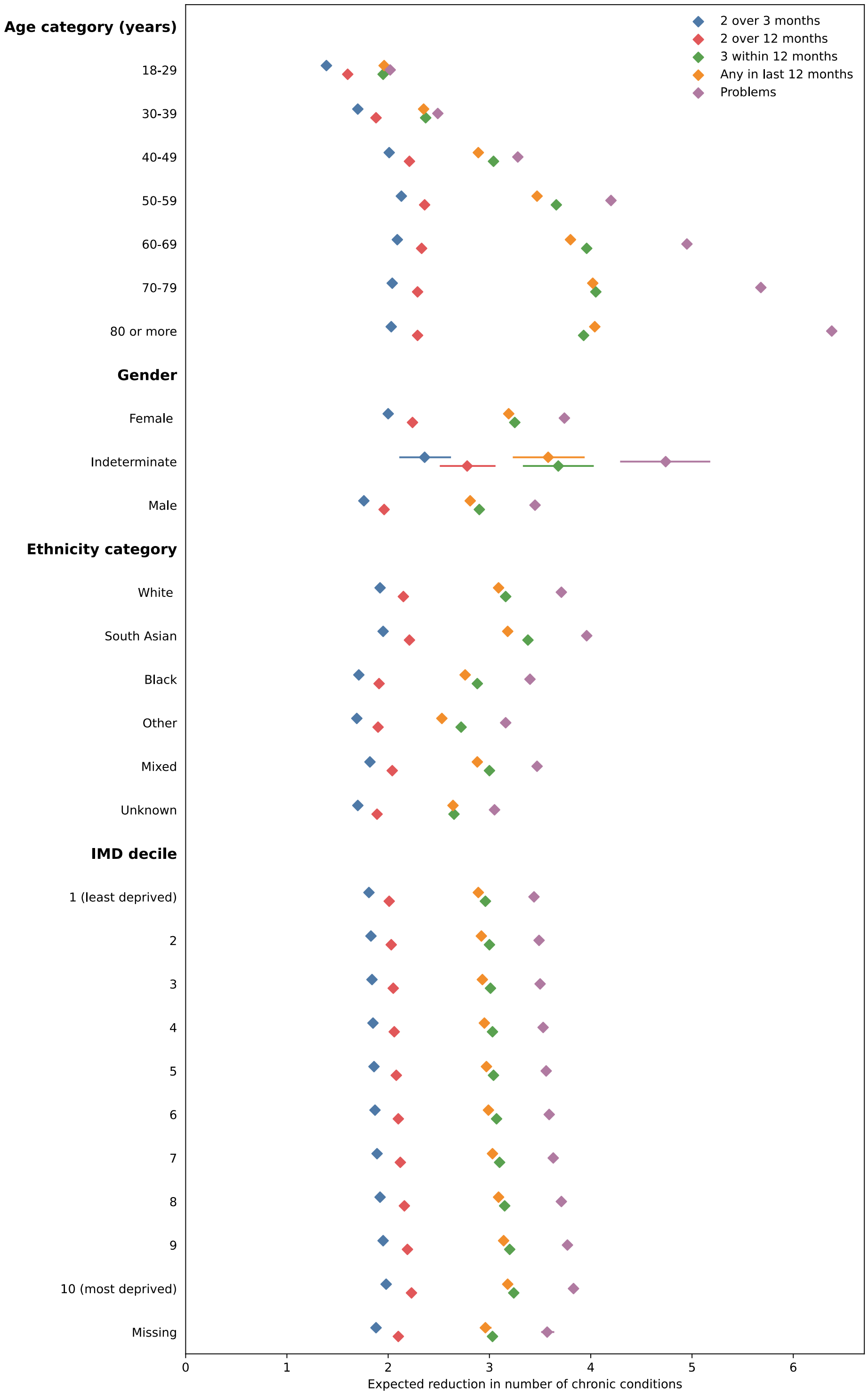

From mixed effects negative binomial regression models. Points represent estimates and bars represent 95% confidence intervals of the estimates.

Table A4: Expected reduction in number of long-term conditions and 95% confidence interval with each definition compared to a single code definition

DEFINITION

VARIABLE	2 over 3		2 over 12		3 within 12		Any in last 12		Problems	
	Coefficient	95% CI	Coefficient	95% CI	Coefficient	95% CI	Coefficient	95% CI	Coefficient	95% CI
**AGE CATEGORY (YEARS)**										
18-29	1.39	1.38 - 1.40	1.60	1.59 - 1.61	1.95	1.94 - 1.96	1.96	1.94 - 1.97	2.02	2.01 - 2.04
30-39	1.70	1.69 - 1.70	1.88	1.87 - 1.89	2.37	2.35 - 2.38	2.35	2.33 - 2.37	2.49	2.47 - 2.51
40-49	2.01	1.99 - 2.02	2.21	2.20 - 2.22	3.04	3.02 - 3.05	2.89	2.87 - 2.92	3.28	3.26 - 3.31
50-59	2.13	2.12 - 2.14	2.36	2.35 - 2.37	3.66	3.64 - 3.68	3.47	3.44 - 3.50	4.20	4.16 - 4.23
60-69	2.09	2.08 - 2.10	2.33	2.32 - 2.35	3.96	3.94 - 3.99	3.80	3.77 - 3.84	4.95	4.91 - 4.98
70-79	2.04	2.03 - 2.05	2.29	2.28 - 2.31	4.05	4.03 - 4.08	4.02	3.99 - 4.06	5.68	5.64 - 5.73
80 or more	2.03	2.02 - 2.04	2.29	2.27 - 2.30	3.93	3.91 - 3.96	4.04	4.00 - 4.07	6.38	6.33 - 6.43
**GENDER**										
Female	2.00	1.99 - 2.01	2.24	2.23 - 2.25	3.25	3.23 - 3.27	3.19	3.17 - 3.22	3.74	3.71 - 3.77
Indeterminate	2.36	2.11 - 2.62	2.78	2.51 - 3.06	3.68	3.33 - 4.03	3.58	3.23 - 3.94	4.74	4.29 - 5.18
Male	1.76	1.75 - 1.77	1.96	1.95 - 1.97	2.90	2.88 - 2.91	2.81	2.79 - 2.83	3.45	3.42 - 3.48
**ETHNICITY CATEGORY**										
White	1.92	1.91 - 1.93	2.15	2.14 - 2.16	3.16	3.14 - 3.18	3.09	3.07 - 3.11	3.71	3.68 - 3.73
South Asian	1.95	1.94 - 1.96	2.21	2.20 - 2.22	3.38	3.36 - 3.40	3.18	3.16 - 3.21	3.96	3.93 - 3.99
Black	1.71	1.70 - 1.72	1.91	1.90 - 1.92	2.88	2.87 - 2.90	2.76	2.74 - 2.78	3.40	3.37 - 3.43
Other	1.69	1.68 - 1.70	1.90	1.89 - 1.91	2.72	2.70 - 2.74	2.53	2.51 - 2.55	3.16	3.13 - 3.18
Mixed	1.82	1.81 - 1.84	2.04	2.03 - 2.06	3.00	2.98 - 3.02	2.88	2.85 - 2.90	3.47	3.44 - 3.50
Unknown	1.70	1.69 - 1.70	1.89	1.88 - 1.90	2.65	2.63 - 2.66	2.64	2.62 - 2.66	3.05	3.03 - 3.07
**IMD DECILE**										
1 (least deprived)	1.81	1.80 - 1.82	2.01	2.00 - 2.02	2.96	2.95 - 2.98	2.89	2.86 - 2.91	3.44	3.41 - 3.47
2	1.83	1.82 - 1.84	2.03	2.02 - 2.04	3.00	2.98 - 3.01	2.92	2.90 - 2.94	3.49	3.46 - 3.51
3	1.84	1.83 - 1.85	2.05	2.04 - 2.06	3.01	2.99 - 3.02	2.93	2.91 - 2.96	3.50	3.47 - 3.53
4	1.85	1.84 - 1.86	2.06	2.05 - 2.07	3.03	3.01 - 3.04	2.95	2.92 - 2.97	3.53	3.50 - 3.55
5	1.86	1.85 - 1.87	2.08	2.06 - 2.09	3.04	3.03 - 3.06	2.97	2.94 - 2.99	3.56	3.53 - 3.58
6	1.87	1.86 - 1.88	2.10	2.09 - 2.11	3.07	3.05 - 3.09	2.99	2.97 - 3.02	3.59	3.57 - 3.62
7	1.89	1.88 - 1.90	2.12	2.11 - 2.13	3.10	3.08 - 3.12	3.03	3.01 - 3.05	3.63	3.61 - 3.66
8	1.92	1.91 - 1.93	2.16	2.15 - 2.17	3.15	3.13 - 3.17	3.09	3.06 - 3.11	3.71	3.68 - 3.74
9	1.95	1.94 - 1.96	2.19	2.18 - 2.20	3.20	3.18 - 3.21	3.14	3.11 - 3.16	3.77	3.74 - 3.80
10 (most deprived)	1.98	1.97 - 1.99	2.23	2.22 - 2.25	3.24	3.22 - 3.26	3.18	3.16 - 3.21	3.83	3.80 - 3.86
Missing	1.88	1.84 - 1.91	2.10	2.06 - 2.14	3.03	2.98 - 3.08	2.96	2.91 - 3.02	3.57	3.51 - 3.64

References

1. Davidson, J. *et al.* Codelists for: ‘Ethnic differences in the incidence of clinically diagnosed influenza: an England population-based cohort study 2008-2018’. https://datacompass.lshtm.ac.uk/id/eprint/2102/ (2021) doi:10.17037/DATA.00002102.
2. Mathur, R., Palla, L., Farmer, R. E., Chaturvedi, N. & Smeeth, L. Ethnic differences in the severity and clinical management of type 2 diabetes at time of diagnosis: A cohort study in the UK Clinical Practice Research Datalink. *Diabetes Research and Clinical Practice* **160**, (2020).
3. Head, A. *et al.* Inequalities in incident and prevalent multimorbidity in England, 2004–19: a population-based, descriptive study. *The Lancet Healthy Longevity* **2**, e489–e497 (2021).
4. Kuan, V. *et al.* A chronological map of 308 physical and mental health conditions from 4 million individuals in the English National Health Service. *The Lancet Digital Health* **1**, e63–e77 (2019).
5. Bisquera, A. *et al.* Inequalities in developing multimorbidity over time: A population-based cohort study from an urban, multi-ethnic borough in the United Kingdom. *Lancet Reg Health Eur* **12**, 100247 (2021).
6. Ashworth, M. *et al.* Journey to multimorbidity: longitudinal analysis exploring cardiovascular risk factors and sociodemographic determinants in an urban setting. *BMJ Open* **9**, (2019).
7. Bhaskaran, K., Forbes, H. J., Douglas, I., Leon, D. A. & Smeeth, L. Representativeness and optimal use of body mass index (BMI) in the UK Clinical Practice Research Datalink (CPRD). *BMJ Open* **3**, e003389 (2013).
